# The diagnosis and management of mucopolysaccharidosis type II

**DOI:** 10.1186/s13052-024-01769-9

**Published:** 2024-10-08

**Authors:** Shao-Jia Mao, Qing-Qing Chen, Yang-Li Dai, Guan-Ping Dong, Chao-Chun Zou

**Affiliations:** Department of Endocrinology, Children’s Hospital of Zhejiang University School of Medicine, Hangzhou, China

**Keywords:** Mucopolysaccharidosis type II, Glycosaminoglycans, Enzyme replacement therapy, Hematopoietic stem cell transplantation, Substrate reduction therapy, Gene therapy

## Abstract

Mucopolysaccharidosis type II (MPS II) is a rare X-linked recessive inherited lysosomal storage disease. With pathogenic variants of the *IDS* gene, the activity of iduronate-2-sulfatase (IDS) is reduced or lost, causing the inability to degrade glycosaminoglycans (GAGs) in cells and influencing cell function, eventually resulting in multisystemic manifestations, such as a coarse face, dysostosis multiplex, recurrent respiratory tract infections, and hernias. Diagnosing MPS II requires a combination of clinical manifestations, imaging examinations, urinary GAGs screening, enzyme activity, and genetic testing. Currently, symptomatic treatment is the main therapeutic approach. Owing to economic and drug availability issues, only a minority of patients opt for enzyme replacement therapy or hematopoietic stem cell transplantation. The limited awareness of the disease, the lack of widespread detection technology, and uneven economic development contribute to the high rates of misdiagnosis and missed diagnosis in China.

## Epidemiology

The incidence of mucopolysaccharidosis type II (MPS II, Hunter syndrome, OMIM 309900) varies by region and ethnicity [1]. According to the literature, MPS II has the highest incidence among all types of MPS in Brazil, Japan, and South Korea, with rates of 0.48/100,000, 0.84/100,000, and 0.74/100,000 live births, respectively, accounting for 29.84%, 55%, and 54.6% of the total number of diagnosed MPS cases in these countries [1–3]. In general, MPS II is the most common type of MPS in East Asia. However, in Mexico, the incidence rate of MPS II is the lowest among all types, at only 0.15/100,000 live births, accounting for 6.76% of the total confirmed number of MPS cases [4]. The majority of patients are male, with a total incidence rate of approximately 1/160,000 live male births [5]. A few female cases have also been reported, mainly due to nonrandom inactivation of the paternal X chromosome [6–9].

## Pathogenesis

MPS II is an X-linked recessive genetic disease caused by pathogenic variants in the *IDS* gene (OMIM 300823) located on chromosome Xq28. The gene spans approximately 44 kb, contains 9 exons, and encodes iduronate-2-sulfatase (IDS), a 550-amino acid enzyme that participates in the first step of the degradation of dermatan sulfate (DS) and heparan sulfate (HS) in lysosomes [5, 6, 10]. Pathogenic variants of the *IDS* gene reduce and abolish enzyme activity, preventing the breakdown of glycosaminoglycans (GAGs) and leading to their gradual accumulation in lysosomes. This interferes with various cell functions, such as cell adhesion, endocytosis, and transport, resulting in multiple organ dysfunction [5, 6]. According to the human gene mutation database (HGMD) professionals, 835 different pathogenic variants have been reported as of January 2023 (HGMD^®^ gene result (cf.ac.uk)), most of which are missense mutations or nonsense mutations, and the rest include splice site mutations and frameshift mutations [10]. There is a correlation between genotype and phenotype; for example, small mutations tend to result in a milder phenotype, whereas large deletions or mutations tend to cause severe phenotypes, such as nonsense mutations, frameshift mutations, and splice site mutations [6, 11]. On the basis of three published genotype cohorts of Chinese MPS II patients, c.1122 C > T (p.Gly374Gly), c.1472 C > A (p.Ser491Tyr), and codon 468 may be common mutation sites in China [11–13]. According to the literature, the RNA expression level of the *IDS* gene was detected in 27 tissues of the human body, with the highest expression level in the brain (Tissue expression of IDS - Summary - The Human Protein Atlas), so pathogenic variants of the *IDS* gene could easily affect the normal function of the nervous system [14].

## Clinical features

MPS II has traditionally been roughly divided into two forms: mild and severe. Both forms present with multiple organ dysfunctions, such as a coarse face, dysostosis multiplex, recurrent respiratory infections, organomegaly, hernias, Mongolian spots, short stature, cardiovascular disease, and hearing impairment [15]. The classification of the two forms is mainly based on the presence or absence of central nervous system (CNS) involvement and the length of survival [16]. The severe form accounts for approximately two-thirds of cases and is often characterized by early onset and rapid disease progression, with onset ages ranging from 18 to 36 months and a mean onset age of 2.47 years. Nonetheless, other studies also document instances where severe cases can manifest within the first year of an individual’s life. These patients have CNS involvement, which manifests mainly as progressive cognitive impairment, with a short life expectancy, and often die from severe respiratory disease or cardiac disease before the age of 20 years. In contrast, the mild form has a relatively late onset age, generally delayed by approximately 2 years, with an average onset age of 4.30 years. The disease progression is relatively slow, and patients generally do not exhibit obvious cognitive impairment and may survive to middle age [6, 17–20]. However, further studies revealed that the phenotype spectrum may change over time and that neurological symptoms may gradually emerge in the later stage. Therefore, some patients may not fit well into the mild or severe classification and should be regarded as a continuum between mild and severe [16, 17]. The primary symptoms, which are outlined below, are as follows:

### Head and face

Some patients develop macrocephaly due to thickening of the skull [11]. Most patients do not have obvious facial abnormalities at birth, but they gradually develop coarse faces with age, which are characterized mainly by a prominent forehead, thick hair, ruddy cheeks, a flat nasal bridge, a blunt nasal tip, and thick lips [21].

### Eyes

As they age, some patients gradually develop lens opacity, reduced visual acuity, amblyopia, refractive errors (such as myopia, hyperopia, and astigmatism), nyctalopia, ocular hypertension, glaucoma, optic neuropathies (such as optic nerve atrophy and optic disc swelling), retinal pigment epithelial changes, delayed visual evoked potentials, exophthalmos, and sclera thickening [22, 23]. Corneal clouding is a prominent feature of MPS I, IVA, VI, and VII but is rare in MPS II [23, 24]. In most patients, visual impairment progresses slowly and irreversibly, but some patients experience rapid vision loss due to optic nerve swelling [22].

### Ear-nose-throat (ENT) and mouth

The deposition of GAGs in tissue causes patients to present multiple corresponding symptoms. Common ENT symptoms include recurrent ear infections, rhinosinusitis, conductive or sensorineural hearing impairment, airway obstruction, tonsil/adenoid hypertrophy, snoring, rhinorrhea, restricted mouth opening due to limited movement of the temporomandibular joint, hoarseness, swallowing disorders, etc [6, 25–28]. Common oral symptoms include macroglossia, tongue extension, gingival hyperplasia, dental caries, periodontal disease, a wide flat palate, an irregular tooth shape, etc [29].

### Bones and joints

The excessive accumulation of GAGs in connective-tissue-forming cells (such as mesenchymal cells, osteoblasts, osteoclasts, and chondrocytes) causes dysostosis multiplex, and most patients exhibit dwarfism characterized by a short trunk and sternal malformation [30, 31]. Other common symptoms include joint deformities (e.g., joint stiffness, contracture, and enlargement), spinal deformities (e.g., scoliosis and kyphosis), pectus carinatum, hip dysplasia, genu valgum, walking on tiptoes, bone pain, claw hands, and triggering fingers [32]. X-rays often reveal wide oar-shaped ribs and bear-like vertebrae.

### Nervous system

The neurological symptoms of patients with a severe phenotype gradually develop with age. At birth, there is typically no notable disparity in appearance or usual health indicators between patients and normal newborns. However, over time, patients show developmental delays in motor, language, cognitive and other aspects compared with their peers. One study revealed that patients reached peak ability between the ages of 5 and 7 years, followed by regression, with more impairments in fine motor rather than gross motor skills [33]. With the worsening of cognitive impairment, patients often exhibit behavioral abnormalities, such as hyperactivity, a lack of concentration, and aggressive behaviors [33, 34]. Some patients have symptoms such as quadriplegia, cerebral palsy, and neurogenic bladder disease due to spinal cord compression. Other common clinical manifestations include disturbed sleep, epilepsy, carpal tunnel syndrome, communicating hydrocephalus, and sustained chewing [35–37]. One previous case reported cerebral infarction in a patient with MPS II [35, 38].

### Digestive system

Recurrent umbilical or inguinal hernias and unexplained chronic watery diarrhea are common complications in some patients [39]. The accumulation of GAGs in organs results in evident hepatosplenomegaly, which manifests as abdominal distension but usually does not impair liver or spleen function. As time progresses, patients often experience constipation due to decreased mobility and muscle weakness [16].

### Cardiovascular system

In Brazil, progressive cardiovascular disease is observed in 82% of patients [40, 41]. The most prevalent phenotype is cardiac valve disease, which often presents as thickening, regurgitation or stenosis of the aortic and mitral valves, resulting in ventricular hypertrophy and heart failure. Other manifestations include aortic root dilatation, cardiomyopathy, arrhythmia, and hypertension [40, 42, 43].

### Respiratory system

The accumulation of GAGs in the mouth, throat, trachea, and other respiratory tissues is prone to cause adenoid/tonsil hypertrophy, tracheomalacia, and increased secretion, which results in progressive airway obstruction and dysfunction. These are important factors for sleep apnea syndrome, frequent upper respiratory tract infections and pneumonia [28, 44–46]. Symptoms such as chest wall deformities and hepatosplenomegaly impair normal contraction and relaxation of the lungs, increasing the risk of respiratory infections. Heart failure can also cause pneumonitis [44, 45].

### Growth and development

According to the literature, most patients are taller and heavier than their healthy peers are in the early stage of growth and development, with a faster growth rate from birth to 1 year of age. As they age, the growth rate slows down or even stops. The characteristic growth pattern in MPS II can be described as excessive growth before the age of 4 years and deceleration or stagnation after approximately 6 years of age. The weight growth rapidly increased in the early stage and gradually decreased in the later stage. The cause of short stature in patients with this type is unclear and may be related to the disruption caused by GAGs accumulation in osseous growth plates [47–49].

### Skin

A network of pebble-like white papules and nodules can be observed on the body, often located on the scapula, back, chest, four limbs, etc [30, 50]. The blue‒gray or blue‒green Mongolian spots have been widely distributed on the body of most patients since birth; these spots are characterized by large numbers, large areas and difficulty in regression and are mainly distributed on the buttocks and back but also on the four limbs, chest, etc [50, 51]. We believe that extensive Mongolian spots can be used for early screening, especially in developing countries where urinary GAG screening and enzyme activity testing are rare. Some patients present with hypertrichosis, with relatively thick and tight skin on their hands [50, 52].

## Diagnosis

Pediatricians may first perform urinary GAGs screening on patients who are suspected of having MPS II to achieve the purpose of preliminary assessment. If the results of urinary GAGs detection are suspicious or positive, further enzyme activity detection or genetic analysis will be performed to confirm the diagnosis. However, it is worth noting that this method also has the possibility of false negatives, and pediatricians need to combine it with clinical judgment (Table 1).

**Table 1:** Diagnostic methods for MPS II

Diagnostic method		Detection principle	Advantage	Disadvantage
Urinary GAGs screening	The quantitative DMB test	Negatively charged GAGs specifically bind with DMB to produce insoluble GAGs dye complexes. Quantitatively analyze the content of GAGs in the dissociation solution by measuring the wavelength with a spectrophotometer.	Quantitative, economical, sensitive, simple and convenient.	Its specificity remains low and it fails to distinguish between different types of MPS. A major limitation is its inability to detect increased GAGs levels of MPS IVA patients.
	LC‒MS/MS	The sample is ionized, accelerated by an electric field into a mass-to-charge ratio selector, and separated by a magnetic field or electric field according to mass and charge.	Sensitive, accurate, capable of distinguishing all types of GAGs and MPS.	Time-consuming, high application cost, high technical difficulty.
Enzyme activity detection	Fibroblasts from skin	Detecting enzyme activity can distinguish MPS subtypes.	For a definite diagnosis	Invasive operation
	White blood cells from blood			High requirements for temperature, transportation and storage
	Collection of dried blood spot samples		Convenient collection	Enzyme activity is easily affected by temperature and transportation time
Molecular genetic testing		The mutated gene loci are diverse in different MPS types.	Easy to collect, helpful for phenotype prediction	Some mutations cannot be detected, such as intron mutation by WES.
NBS		MS or fluorescence assay is generally used to determine the enzyme activity in DBS.	Early diagnosis	Large screening sample size and high application cost.

MPS, mucopolysaccharidosis; GAGs, glycosaminoglycans; WES, whole-exon sequencing; DMB, dimethyl methylene blue; LC‒MS/MS, liquid chromatography tandem mass spectrometry; NBS, newborn screening; DBS, dried blood spot

### Urinary GAGs screening

In the clinic, urinary GAGs detection frequently serves as a preliminary screening approach for MPS. Owing to the lack of enzyme activity caused by gene pathogenic variants, GAGs in cells cannot be degraded in MPS patients, and some GAGs are excreted in the urine. Therefore, MPS can be preliminarily screened by urinary GAGs detection. Clinical laboratories commonly use the dimethyl methylene blue (DMB) colorimetric method to quantitatively detect GAGs in random urine and calculate the ratio of GAGs to creatinine (GAGs/Cr). The principle is based on the negative charge of GAGs and the positive charge of DMB. Under an acidic environment, the two are specifically combined into a complex and decomposed in the dissociation solution. The content of GAGs is subsequently quantified via a spectrophotometer. The normal range of urinary GAGs/Cr varies with age, and when the ratio increases, it indicates the possibility of MPS. However, this test is affected by renal function, phenotype, and other factors, which may lead to misdiagnosis. Urinary GAGs can also be detected qualitatively via electrophoresis. In recent years, liquid chromatography–tandem mass spectrometry (LC‒MS/MS), which can quantify and distinguish various components of GAGs, has been gradually developed to preliminarily identify the types of MPS [53]. Urinary GAGs are also currently used to evaluate efficacy after enzyme replacement therapy (ERT) or hematopoietic stem cell transplantation (HSCT).

### Enzyme activity detection

Different types of MPS are characterized by mutations in different genes, resulting in the deficiency of distinct enzymes; thus, the detection of enzyme activity can be used to classify and diagnose MPS. Fibroblasts obtained from skin biopsies or white blood cells from peripheral blood, which are currently used as clinical samples, serve for a definite diagnosis. White blood cell samples are susceptible to temperature and have high transportation and storage requirements. Although fibroblasts are not affected by temperature or transport and can be remeasured through cell culture, they are rarely used in clinical practice because they are invasive. Dried blood spot (DBS) samples are relatively convenient to collect, but enzyme activity is easily disturbed by temperature and transportation time, and the number of cells collected through the spot is relatively small. If the DBS test is positive, further molecular testing is recommended to assist in the diagnosis [11, 54].

### Molecular genetic testing

Genetic testing is also an effective diagnostic method for MPS II because of pathogenic variants of the *IDS* gene, which can help differentiate it from other diseases with similar symptoms and further guide couples in cases of future pregnancies [55]. Studies have shown a relationship between genotypes and phenotypes. Detecting mutation sites is helpful for phenotype prediction and can guide early clinical treatment [11]. Currently, whole-genome/exome sequencing (WGS/WES) is commonly used. However, there are certain limitations, such as that WES cannot detect some fragment deletions or duplications, intron mutations, etc [56]. Comparably, WGS or nanopore sequences are more comprehensive in detecting mutation sites. Array-CGH analysis, which excels in detecting fragment genomic deletions/duplications, may assist in diagnosis [57].

### Newborn screening (NBS)

Tandem mass spectrometry or fluorescence assays are generally used to determine enzyme activity in DBS for NBS [58]. The progression of MPS II is progressive, and early diagnosis is very important for improving the prognosis. According to reports, some countries (e.g., the United States) included MPS II in the early NBS program [59]. Neonatal genetic screening using filter paper is being carried out in China, serving as a valuable supplement to biochemical testing and offering promising development potential as a high-throughput screening method.

## Management

With the progress of medical research and medical security systems, a variety of treatments have emerged, such as ERT, HSCT, and gene therapy, which are not limited to palliative treatment. Through multidisciplinary management, delaying progression and even achieving long-term survival seems more than just a dream. Certainly, good family management is also an essential factor in improving the quality of life of patients [60]. Owing to economic and medical limitations, most patients in China still focus on palliative treatment (Table 2).

**Table 2:** Treatment methods for MPS II

Treatments	Treatment Principle	Advantage	Disadvantage
Palliative treatment	Patients may experience multisystem symptoms, and palliative treatment can alleviate their suffering.	Relatively low price and high accessibility.	Cannot halt the progression of the disease.
ERT	Recombinant IDS enzyme injected intravenously enter the cell through endocytosis mediated and are transported to lysosomes for catabolism of accumulated substrates	Compared with HSCT, ERT is safer, simpler to operate, and can delay the progression of the disease.	Expensive price, requiring long-term infusion, limited improvement in certain symptoms.
HSCT	Making the enzyme-producing donor cells enter the blood circulation. Through cross-correction, the enzymes enter the recipient cells and correct the substrate accumulation.	Producing enzymes continuously, can improve neurological symptoms and at a lower relative medical cost.	HSCT carries certain risks, including postoperative infection, transplant rejection, graft-versus-host disease.
Gene therapy	Introducing the target gene into the target cells through the vector to correct the defect of not producing IDS enzyme in vivo.	Improving neurological symptoms, delaying the progression, and even curing the disease.	Not yet applied to clinical practice, safety unknown.
SRT	Inhibiting the production of GAGs, thereby reducing intracellular substrate accumulation.	This therapy is applicable to all types of MPS and can cross the BBB.	Further clinical trials have not been conducted to verify its effect on MPS.

MPS, mucopolysaccharidosis; GAGs, glycosaminoglycans; ERT, enzyme replacement therapy; HSCT, hematopoietic stem cell transplantation; SRT, substrate reduction therapy; BBB, blood–brain barrier

### Palliative treatment

Patients often experience progressive multisystem symptoms, which require the active participation of various departments in treatment to reduce their suffering [61]. For example, orthopedic surgeons may use braces or surgical corrections to help patients potentially slow bone deformities and maintain motor function, contingent upon the extent and nature of their condition. Endocrinologists may prescribe somatropin therapy for patients with short stature; although research suggests that growth hormone has no significant beneficial effects on patients’ growth rate, it can increase bone density and reduce fat mass [62]. Symptoms such as hydrocephalus and spinal cord compression can be alleviated by surgical decompression. However, nervous system symptoms cannot be completely cured, as drugs cannot cross the blood–brain barrier (BBB) [10]. In summary, palliative treatment currently mitigates symptoms but does not halt the progression of the disease.

### ERT

The recombinant IDS enzyme injected intravenously enters the cell through endocytosis mediated by mannose 6-phosphate receptor (M6P-R) proteins and is transported to lysosomes for catabolism of accumulated substrates. Animal and clinical trials have shown that intravenous infusion of recombinant enzymes can significantly reduce urinary GAGs, decrease the volume of the liver and spleen, and improve the 6-minute walking test score, growth and lung function [63–66]. However, its effects on tracheal deformities, skeletal joints, hearing loss, the nervous system (such as cognitive impairment and language developmental disorders), and heart valves are limited [67–70]. The most common adverse reaction to this therapy is an infusion-related hypersensitivity reaction, which mainly manifests as rash, fever, and headache and can be managed by slowing the infusion rate and using antihistamines [19, 71, 72]. The safety of ERT has been demonstrated in decades of research, with fewer risks and simpler procedures than those of HSCT, but the ERT enzyme has a short half-life and requires regular injections. In the early 21st century, the recombinant IDS enzyme was approved in the United States and Europe for clinical use [19]. Studies have indicated that approximately half of patients receiving ERT develop related antibodies during treatment, which may affect the efficacy of ERT [71, 73]. As some symptoms are irreversible, the earlier ERT is used, the more beneficial the prognosis of patients will be [74, 75]. Clinicians should be careful not to discontinue drug application abruptly after initiating treatment, as this may lead to rapid deterioration of clinical symptoms [76, 77].

To overcome the limitation that recombinant enzymes cannot cross the BBB and do not improve neurological symptoms, researchers have developed methods to enable recombinant enzymes to penetrate the BBB. One method involves regular intracerebroventricular injection of drugs. Mouse experiments have demonstrated that this method can reduce the level of substrate accumulation in the brain and significantly improve learning/memory function [78]. A clinical study showed that intrathecal injection of different doses of recombinant enzyme significantly decreased the level of GAGs in the brains of patients [73]. Another method involves the recombinant enzyme and the corresponding antibody to form fusion proteins, which are targeted to bind the insulin receptor or transferrin receptor and facilitate the entry of the recombinant enzyme into the BBB through receptor-mediated endocytosis [79]. A phase 1/2 clinical trial showed that weekly intravenous injection of the IDS enzyme combined with an anti-human transferrin receptor antibody (JR-141, JCR Pharmaceuticals, Japan) significantly reduced the levels of HS and DS in cerebrospinal fluid, indicating the efficacy of the treatment in the brain [80, 81]. A phase 2/3 trial of Pabinafusp Alfa (JR-141) further demonstrated a significant decrease in HS in cerebrospinal fluid, as well as in the serum HS concentration and liver and spleen volume [82].

### HSCT

HSCT can cause enzyme-producing donor cells to enter the blood circulation. Once engrafted, these cells provide a sustained source of enzymes. Through cross-correction, the enzymes enter the recipient cells and correct substrate accumulation [83, 84]. One study revealed that donor-derived microglia were detected in patients’ brains after HSCT, suggesting that HSCT may improve neurological symptoms [85, 86]. Compared with ERT, HSCT produces enzymes more continuously, does not require regular supplementation, and can cross the BBB to produce enzymes in brain tissue, improving neurological symptoms and lowering the relative medical cost. Studies have shown that symptoms such as vision, hearing, hepatosplenomegaly, lung function, and a coarse face improve after HSCT, but this improvement is not obvious in bones, heart valves, or the damaged nervous system because of relatively few blood vessels and limited enzyme production by donor cells [6, 87]. A study from Japan showed that when HSCT was used in patients who had not yet developed brain atrophy or heart valve involvement, it had a protective effect on the subsequent brain and heart [88]. If neurodevelopmental delay has occurred before transplantation, symptoms cannot be significantly improved by HSCT, so early treatment is recommended for patients with mild organ involvement [87]. There are certain risks associated with HSCT, including postoperative infection, transplant rejection, and graft-versus-host disease. Before treatment, doctors and families should jointly evaluate the risks and benefits.

### Gene therapy

As MPS II is a monogenic disease with a relatively clear pathogenesis, it is a suitable target for gene therapy. An in vivo study revealed that cocultured fibroblasts from MPS II patients and lymphoblastoid cell lines transduced with an amphotropic retroviral vector with the *IDS* gene exhibit GAG degradation, which is a significant basis for supporting the potential effectiveness of gene therapy [89, 90]. The basic principle is to deliver a functional gene into specific cells via a vector system, aiming to compensate for the in vivo deficiency in the production of the IDS enzyme. Vectors can be classified into viral and nonviral types. The viral type includes retrovirus, lentivirus, and adeno-associated virus, and the nonviral type includes the Sleeping Beauty transposon system and nanovectors. Gene therapy can be divided into two methods: in vivo and in vitro. In vivo therapy involves the direct injection of a vector with the desired gene into the body. Intracranial targeted stem cell gene therapy is based on an adeno-associated virus as a carrier, and the virus carrying the *IDS* gene is directly injected into the cerebrospinal fluid. A mouse model study revealed significant increases in enzyme activity in the central nervous system and improvements in cognitive and motor function. Relative clinical trials are in progress [15]. In in vitro therapy, the target gene is introduced into the obtained recipient cells in vitro, and then, the modified cells are transfused back into the body. Hematopoietic stem cell gene therapy (HSCGT) is the primary treatment modality, utilizing the proliferative potential of hematopoietic stem cells. After complete bone marrow clearance, autologous CD_34_^+^ peripheral blood stem cells are transduced in vitro via lentiviral vectors as carriers. The genetically modified CD_34_^+^ cells are subsequently reintroduced into patients. Compared with HSCT, HSCGT has obvious advantages. It does not need to find a matching donor and has a low risk of immune rejection. Animal experiments have shown that after HSCGT treatment, superphysiological enzyme activity is detected in most peripheral tissues, which demonstrates the potential efficacy of this treatment [84, 91].

### Others

Substrate reduction therapy (SRT) is designed to inhibit the production of GAGs, thereby reducing intracellular substrate accumulation. Since the substrates of the MPS are the same across different types, SRT is suitable for all types and can cross the BBB. SRT has been successfully applied to lysosomal storage diseases such as Fabry disease and Gaucher disease [92]. Studies have shown that genistein can suppress GAGs synthesis and improve the cell cycle of MPS II by inhibiting the phosphorylation of epidermal growth factor receptor and subsequently affecting the expression of specific genes regulated by EGF-dependent signal transduction pathways [56, 93]. Further clinical observations of MPS II have shown that genistein could improve joint mobility [94]. Rhodamine B, another GAGs synthesis inhibitor, has been shown to improve neurological symptoms in MPS IIIA mice [95]. It has been reported that acute exposure to rhodamine B can harm the human body, and it may be a nonspecific inhibitor of GAGs synthesis; however, further clinical trials have not been conducted to verify its effect on MPS [96].

## Conclusion

MPS II is an X-linked recessive genetic disease with a progressive course. Symptoms mainly appears gradually after birth, and some symptoms are irreversible. Therefore, early screening, diagnosis, and treatment are essential for the prognosis of patients. At present, palliative treatment is still the main treatment in developing countries. ERT and HSCT are also carried out in some patients, but palliative treatment and ERT have not significantly improved the CNS manifestations of patients thus far. Patients with damaged nervous systems benefit little from HSCT. New treatments that can penetrate the BBB, such as gene therapy and SRT, are under active research, which provides hope for severe patients in the future. Ultimately, further research could focus on investigating the efficacy of combination therapy.

## Data Availability

Not applicable.

## Abbreviations

MPS: Mucopolysaccharidosis
GAGs: Glycosaminoglycans
IDS: Iduronate-2-sulfatase
DS: Dermatan sulfate
HS: Heparan sulfate
CNS: Central nervous system
ENT: Ear-nose-throat
WGS: Whole-genome sequencing
WES: Whole-exome sequencing
DBS: Dried blood spot
DMB: Dimethyl methylene blue
LC‒MS/MS: Liquid chromatography–tandem mass spectrometry
ERT: Enzyme replacement therapy
HSCT: Hematopoietic stem cell transplantation
BBB: Blood–brain barrier
M6P-R: Mannose 6-phosphate receptor
HSCGT: Hematopoietic stem cell gene therapy
SRT: Substrate reduction therapy
